# Characterization of Crude and Processed Pulp Cell Walls of Three Selected Mamey Accessions (*Mammea americana* L.)

**DOI:** 10.3390/molecules29071596

**Published:** 2024-04-03

**Authors:** Déborah Palmont, Estelle Bonnin, Emilie J. Smith Ravin, Marc Lahaye, Odile Marcelin

**Affiliations:** 1 Groupe de Recherche BIOSPHERES, Université des Antilles, BP 7209, 97275 Schœlcher, France; deborah.palmont@gmail.com (D.P.); ravinemilie@hotmail.com (E.J.S.R.); 2 INRAE (Institut National de Recherche Pour l’Agriculture, l’Alimentation et l’Environnement), UR BIA Biopolymères Interactions Assemblages, 44316 Nantes Cedex, France; estelle.bonnin@inrae.fr (E.B.); marc.lahaye@inrae.fr (M.L.)

**Keywords:** *Mammea americana* L., mamey pulp, cell wall, pectin, immunolabeling

## Abstract

Mamey (*Mammea americana* L.) is a tropical fleshy fruit native from the West Indies and northern South America. It is very appreciated for its flavor and color but has been little described. The present study investigates the composition and histochemistry of the pulp cell walls of three mamey accessions readily available in Martinique. The impact of pulp processing into puree on cell wall composition is evaluated. The histology and rheology of mamey puree are assessed considering these characterizations. Mamey pulp cell wall composition is dominated by highly methyl-esterified pectins (DM: 66.2–76.7%) of high molecular weight, and show few hemicelluloses, mainly xyloglucans. Processing reduced methyl-esterified uronic acid contents and gave purees with significantly different viscosities. Mamey puree was composed of polydisperse particles (20–2343 µm), which size distributions were different depending on the accession: Ti Jacques was dominated by smaller particles (50% had approximated diameters lower than 160 µm), Sonson’s by larger particles (50% had approximated diameters higher than 900 µm), and Galion’s had an intermediate profile. This new knowledge on mamey pulp is valuable for future works on mamey processing into new food products, even more so for those including cell wall polysaccharide-degrading enzymes.

## 1. Introduction

Mamey (*Mammea americana* L., Callophylaceae) is native from the West Indies and the north of South America, and is now found in Florida as well as of Asia and, more rarely, west Africa [1]. It is among the tropical fruits that are widely appreciated for their flavor, color and nutritional benefits. The fruit is mainly consumed fresh, the pulp processed into juice and jam, and the flowers are used in a flavored liquor. Mamey is a berry with a thick leathery russet to grayish-brown peel, a thin whitish-yellow layer that covers the yellow to orange flesh and one to four seeds on average, with variable size and weight [2]. The whole berry can weigh from 0.5 kg up to 2 kg and its pulp is reported to represent about 60 to 70% of the whole weight [2,3]. Mamey pulp has been particularly recognized for its carotenoid content [4,5,6].

With consumers growing demands for healthy food, mamey has attracted high interest from the food industries. Physicochemical characteristics and processing ability have been recently studied [7,8,9,10,11] in the search for alternative solutions to extend the consumption time of this climacteric fruit. Yet, a better knowledge of the composition, properties and processability of this fruit would promote the development of a brand new food product meeting consumer expectations.

The present study reports the first account of mamey pulp histochemistry and cell wall composition, and the impact of its processing into puree on cell wall composition and rheology. Puree viscosity is analyzed with consideration to histological characteristics. The study focuses on three West Indian accessions found in Martinique named Galion, Ti Jacques and Sonson. These were selected out of eight accessions for their best fruit/puree yields and puree physicochemical characteristics [9]. The new information reported on mamey pulp cell walls contributes to basic knowledge on this tropical fruit and provides ground for its further enzymatic processing into juice.

## 2. Results and Discussion

### 2.1. Characterization of the Cell Walls from Mamey Fruit Pulp

The cell wall polysaccharides from mamey pulp were studied for their chemical composition, structure and distribution in the different tissues.

#### 2.1.1. Cell Wall Yields and Composition

AIR (alcohol-insoluble residue) yield, corresponding fairly well to the cell wall content, was the highest in Ti Jacques fresh pulp and puree (Table 1). Cell wall content was not significantly impacted by processing as AIR yield was comparable in fresh pulp and puree samples for Ti Jacques and Sonson accessions. However, the AIR yields of Galion’s fresh pulp and puree samples were significantly different. It was attributed to material loss during an additional manipulation needed, due to blocking during filtration. The yield ranges were higher than those reported for mango (1.4 ± 0.1 to 1.7 ± 0.1 g/100 g) [12] and guava purees (1.6 g/100 g) [13].

Protein constituted the third main component after uronic acids and cellulosic glucose (Table 1). Protein contents were slightly higher in puree compared to fresh pulp, most likely due to a better extraction of proteins through crushing. Ash content was in the range of 1–2%. Lower protein contents were reported for AIR from guava (5.8 g/100 g) and mango purees (2.5 ± 0.3 to 5.3 ± 0.4 g/100 g), and comparable ash contents were reported for mango purees (ash content: 0.9 ± 0.1 to 1.3 ± 0.1 g/100 g) [12,13].

The three accessions gave rather close results in their sugar contents (Table 1). Slight variations between accessions may be related to the composition of pectin and/or hemicelluloses. An influence of the maturity stage should also be considered as the usual sampling method based on sensorial parameters may induce slight variations between the fruits used to prepare the purees. Uronic acids were the major sugar component in mamey AIR, with twice more UAs than cellulosic glucose. UA contents in puree were slightly lower than in fresh pulp, indicating a slight loss of UAs during transformation. Sonson accession presented higher UA contents than the other two accessions and the lowest loss during transformation (about 3%). It was Ti Jacques that lost the most UAs during transformation (about 10%, Galion: about 6%). The UA-to-rhamnose molar ratio (UA/Rha) gives information on the homogalacturonan (HG)/rhamnogalacturonan (RGI) ratio in the pectin. Sonson UA/Rha ratios (fresh pulp: 32.6 ± 0.2; puree: 23.5) was the highest compared to Galion (fresh pulp: 23.2 ± 2.1; puree: 19.9) and Ti Jacques (fresh pulp: 23.4 ± 1.3; puree: 16.6), suggesting that Sonson pectin contained more homogalacturonan than pectin in the other two accessions. Purees UA/Rha ratios were lower than that of fresh pulp, due to their lower UA contents. The degree of methyl-esterification of UAs in AIR was the highest in Sonson (fresh pulp: 76.7 ± 0.2; puree: 72.1 ± 1.6), suggesting a higher content in highly methylated pectin in this accession. For all accessions, fresh pulp DM was higher than in puree, indicating that the UAs lost during transformation had considerable amounts of methyl esters. The acetic acid contents were comparable in fresh pulp and puree and close between accessions. The arabinose content was higher than the galactose content, suggesting that the pectin side chains were mainly formed of arabinose. Galactose content of Sonson puree AIR was higher than in fresh pulp, confirming the slight over-ripeness observed in one of these fresh fruits as it has been reported that galactose content decreases during fruit ripening [14,15,16]. Except for this case, no significant difference in neutral sugar contents were observed between fresh pulp and puree AIR.

Mamey cell walls exhibited low contents in xylose and mannose, suggesting that they were poor in hemicelluloses compared to guava, in which higher xylose, mannose, fucose and non-cellulosic glucose contents were reported (7.5%, 2.5%, 0.9%, and 4.8%, respectively) [17]. It appeared that mamey cell wall composition was comparable with that of plum with low xylose, mannose, rhamnose, fucose and non-cellulosic glucose contents (about 2.7%, 1.2%, 0.7%, 0.3% and 0.7%, respectively), and high DM and UA contents (about 70% and 27%, respectively) [18]. Similarities were also found with peach and apricot, where the AIR was reported with arabinose (about 45%) and galactose (about 20%) as the main neutral sugars and fucose (about 2.5%), mannose (about 4% and 3%, respectively) and rhamnose (about 6% and 4%, respectively) as the least neutral sugars [19].

#### 2.1.2. Yields and Sugar Composition of Water-Soluble and -Insoluble Polysaccharides

Soluble (WSF) and insoluble (WAIR) fractions were extracted and analyzed for technological purposes in order to guide the further processing strategies of mamey pulp, including cell wall-degrading enzymes.

WSF yields were higher for Galion and Sonson accessions (about 20% of dry weight), indicating that the cell walls from Ti Jacques had lower water-soluble polysaccharides than the others (Table 2). For all accessions, WSF yields were lower than reported for mango (30 to 40% of DW of AIR) [12] and plum [18]. It indicated that mamey cell walls contained less water-soluble polysaccharides than mango and plum cell walls.

In the soluble fraction, UAs were the major sugar, which is consistent with the expected composition of this fraction as water-soluble pectin. UAs from AIR were recovered for about 40% in WSF and 60% in WAIR, where it was the second main component. The AIR pectin recovered in WSF had comparable DM (about 70%) to that of AIR, whereas the pectin recovered in WAIR was less methyl-esterified (about 58%). Therefore, WSF contained the most highly methylated pectin even though methanol was slightly less recovered in WSF (about 34 to 49%) than in WAIR (about 45 to 59%). No systematic significant difference between fresh pulp and puree samples were observed. In contrast to methanol content, acetic acid content did not vary much in WAIR compared to AIR, while it was lower in WSF, corresponding to a degree of acetylation of about 8%. Acetic acid was mainly recovered in the insoluble fraction (about 72 to 79%) compared to the soluble fraction (about 8 to 17%). WSF UA/Rha ratios were about twice as high as AIR ratios (Table 2), indicating an enrichment in HG compared to AIR. WAIR pectin was rather enriched in RGI with ratios UA/Rha twice as low as in AIR. Contrary to AIR, no significant difference in UA/Rha ratios were observed between fresh pulp and puree samples, except for Ti Jacques’ WSF. Therefore, HG and RG regions were equivalently distributed in soluble and insoluble fractions.

Cellulose was recovered in WAIR, where cellulosic glucose was the main sugar. As observed in AIR, arabinose content was higher than galactose and xylose contents in WSF and WAIR. Also, no significant difference in neutral sugar contents were observed between fresh pulp and puree WSF and WAIR, except for galactose in Sonson accession related to one fruit over-ripeness. WAIR had higher xylose, mannose and fucose contents than WSF, indicating a higher amount of hemicellulose in the WAIR.

The determination of the composition in RGI regions and in hemicellulose, mainly consisting of xyloglucan considering hemicellulose-related neutral sugar contents and the fruit origin of the cell walls, is valuable guidance in regard to the wide offer of commercially available enzymes. Enzymatic preparation with pectin-degrading enzymes particularly targeting highly methylated HG, and secondary activities targeting xyloglucan, RGI and arabinan, galactan and arabinogalactan side chains should be preferred.

#### 2.1.3. Yields and Sugar Composition of Water-Soluble and -Insoluble Polysaccharides

WSF were further characterized (Table 3), as they represented about 40% of the AIR pectin and probably impacted the puree texture, particularly its high viscosity.

The three accessions of WSF from fresh pulp samples had comparable Mw, while in puree samples, Mw was the highest in Galion’s WSF and the lowest in Ti Jacques’ WSF. The apple pectin with higher Mw had the lowest intrinsic viscosity (IV) [20], which was not observed here with Ti Jacques puree despite similar polydispersity index and (Ara + Gal)/Rha to Galion. Ti Jacques’ lowest intrinsic viscosity may be related to its lowest UA/Rha ratio that could indicate a higher amount of RGI. Indeed, RGI-rich pectin domains were reported to be more flexible than HG-rich domains [21]. The variability in RGI side chain composition proposed in the previous section may explain the higher polydispersity index observed in Galion and Ti Jacques WSF. The polydispersity index of Sonson was the lowest, indicating that the water-soluble pectin from Sonson was more homogeneous in size than other accessions. (Ara + Gal)/Rha ratio was also higher in Sonson WSF, indicating that RGI was more substituted or had longer side chains compared with the other accessions.

Therefore, WSF from mamey contained high amounts of high-molecular-weight pectin, which differed according to their proportion in RGI and their side chain composition and frequency. These properties contributed to the puree viscosities, forming a network of polymers in puree serum that lubricated these highly concentrated cell suspensions at different levels.

#### 2.1.4. Immunohistochemical Characterization of Cell Wall Polysaccharides

Histological observation was performed in order to characterize the tissues in relation with the physical characteristics of each accession’s flesh.

**Figure 1:**
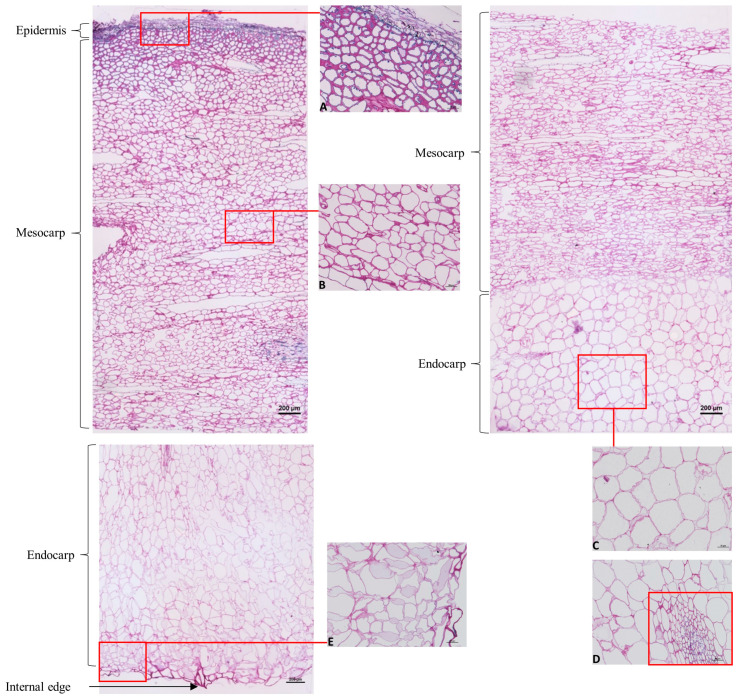
Transverse sections of fresh mamey pulp from Sonson accession stained with toluidine blue. (**A**) Epicarp; (**B**) mesocarp; (**C**) endocarp with vessels (**D**); (**E**) inner region of the fruit pulp, including endocarp and its internal edge. Scale on small frames (**A**–**E**): bar = 50 µm.

Under the conditions used, no autofluorescence was observed in the thin sections of the three accessions studied, allowing us to carry out specific fluorescence assays. Toluidine blue coloration showed clear differences in cell forms and sizes and in cell wall thickness between epidermis/mesocarp and mesocarp/endocarp (Figure 1). Thick cell walls were observed in the epidermis, with small cells (Figure 1A). The mesocarp cells were larger and presented thinner cell walls toward the endocarp area, where they were also more stretched (Figure 1B). Some secretory ducts are observed as previously reported [22]. Areas with non-attached cells were observed in Sonson and Ti Jacques accessions and in a lesser extent in Galion. The endocarp cells had curved shapes with thin cell walls (Figure 1C) and some vascular bundles were observed, as also reported in [22] (Figure 1D). Toward the internal edge of the endocarp, there were areas with non-attached cells mainly in Galion and Sonson, where the cells had irregular shapes (Figure 1E). In contrast, Ti Jacques showed well-defined shapes comparable to that of endocarp cells. The internal edge was thicker in Ti Jacques and Galion compared with Sonson.

**Figure 2:**
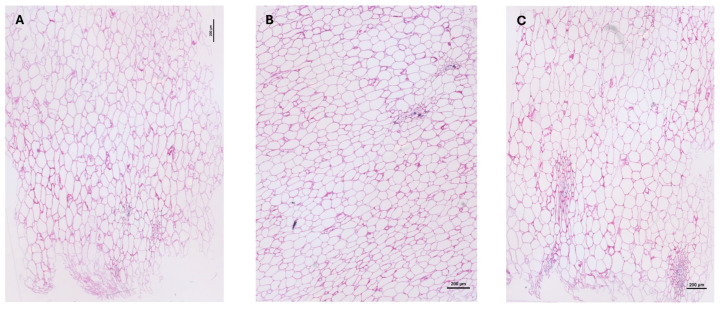
Comparison of transverse sections of fresh mamey endocarp from the three accessions studied stained with toluidine blue. (**A**) Galion; (**B**) Ti Jacques; (**C**) Sonson.

Compared with the other accessions, Sonson mostly had bigger endocarp cells while Ti Jacques accession mostly had smaller endocarp cells (other than vascular bundles) (Figure 2). Galion’s biggest endocarp cells were smaller than Sonson’s and it had fewer small cells than Ti Jacques. These observations are consistent with the fresh pulp AIR contents. Indeed, the smaller the cells, the higher the cell wall content for a given surface.

**Figure 3:**
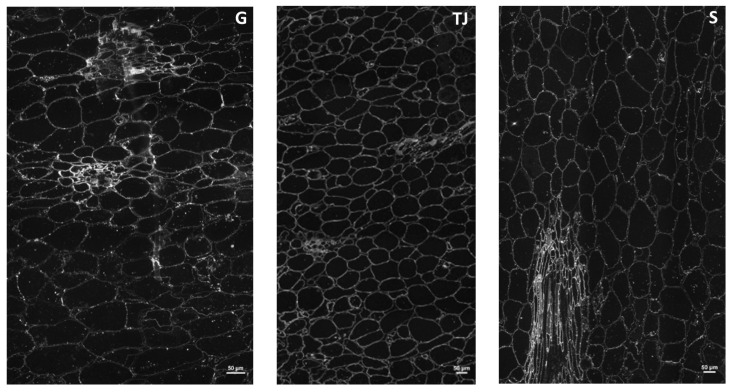
Transverse sections of the endocarp of fresh mamey pulp labeled by CBM3a after pectin lyase and endo-glucanase pretreatment. (G) Galion accession; (TJ) Ti Jacques accession; (S) Sonson accession. Bar = 50 µm.

The endocarp was further studied to see the distribution of the cell wall polysaccharides as it is the edible part that was used to make the puree for previous analyses (Section 2.1.1, Section 2.1.2 and Section 2.1.3). Calcofluor targets β-(1,4) linkages and is specific for non-crystalline cellulose [23]. Using calcofluor on the endocarp semi-thin sections, very weak and heterogeneous fluorescence was detected, indicating low beta-glucan contents. Detection was higher in areas with small cells and globally higher in Sonson accession than in the others. Labeling of the crystalline cellulose with specific CMB3a required a pectin lyase pretreatment, which allowed for detecting fluorescence in all the cell walls with higher fluorescence in vessel cell walls (Figure 3).

Pectin lyase combined with endoglucanase pretreatment did not improve the labeling of crystalline cellulose in Sonson, but slightly improved it in Galion and improved it considerably in Ti Jacques, where detection was the highest. In the case of Sonson, labeling was less homogeneous than with single pectin lyase pretreatment, where it was the highest of all accessions. As CBM3a also targets xyloglucan, and xyloglucan can also be depolymerized by the endoglucanase used for pretreatment, it could indicate that Sonson’s higher labeling detection with single pectin lyase pretreatment was related to the presence of xyloglucan, part of which was lost with endoglucanase pretreatment. The improvement of labeling in Galion and Ti Jacques with the combination of pretreatments rather indicated less xyloglucan and the occurrence of crystalline cellulose masking by pectin and amorphous cellulose, especially for Ti Jacques. Their higher xylose contents compared to Sonson (Table 1) may be related to the presence of glucuronoarabinoxylan or xylogalacturonan. LM19 and LM20 antibodies, specific for weakly and highly methylated HG, respectively, were used to explore pectin in mamey endocarp cell walls. High and homogeneous labeling was observed in all cell walls with LM19 antibodies. With LM20 antibodies, staining appeared weaker and heterogeneous but stronger in vessels’ cell walls. Weaker LM20 staining was observed after pectin lyase pretreatment. This remnant fluorescence detection indicated that pectin lyase pretreatment did not eliminate all the HG detected by LM20 antibodies, although mamey pectin was shown to be highly methylated (Table 1). This suggested the presence of acetyl esters limiting the action of pectin lyase. Using 2F4 antibody, specific for conformational dimeric HG chelated by Ca^2+^, fluorescence was detected in all the cell walls, with higher labeling in the wall lining the intercellular space. It confirmed the presence of tightly cross-linked HG in these zones as already reported in the middle lamella and cell corner regions [24].

High fluorescence was detected in all the cell walls of endocarp cells with INRA-RU1 antibody specific for RGI backbone. After alkaline de-esterification, fluorescence appeared slightly higher, suggesting that RGI carried acetyl esterification. LM5 and LM6 antibodies, specific for galactan and arabinan RGI side chains, respectively, labeled homogeneously the whole cell walls, thereby confirming the presence of pectin neutral sugar side chains in the cell walls of mamey endocarp. LM5 staining was higher than LM6 despite the lower contents of galactose compared to arabinose. It may indicate that arabinan side chains were less accessible than galactan side chains and/or that arabinose residues were implicated in other cell wall components. LM5 staining was slightly higher in Sonson endocarp; this was coherent with its higher (Ara + Gal)/Rha ratio and the hypothesis that its RGI had more side chains. The hemicellulose of mamey endocarp cell walls were probed by LM15 and LM21 antibodies specific for xyloglucan and mannan, respectively. Whatever the pretreatment applied, extremely weak labeling was achieved by LM15 while none was observed with LM21, in agreement with the low hemicellulosic neutral sugar contents. Mannans were scarce in these cell walls and xyloglucans appeared as the main hemicellulose, though at low amounts considering composition analysis (Table 1).

### 2.2. Rheological and Histological Characterization of Mamey Puree

It has been demonstrated that particles’ size and concentration influence rheological behavior of suspensions [25]; therefore, the particle state in the puree was studied.

**Figure 4:**
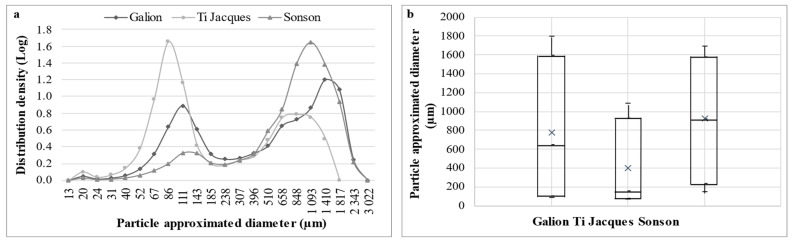
Particle size distribution in Galion, Ti Jacques and Sonson puree. (**a**) Density distribution of particle size. (**b**) Box-plot representation of particle size. Bottom and top limits are the 10th and 90th centiles; inferior and superior sides of the box are the 15th and 85th centiles; the segment inside the box is the median value (50th centile). Average value is represented by the cross inside the box.

Ti Jacques had a significantly higher number of particles analyzed (32.6 × 10^6^ ± 3.4 × 10^6^) than Galion (20.1 × 10^6^ ± 1.9 × 10^6^) and Sonson (12.3 × 10^6^ ± 8.0 × 10^5^) in the same volume of analysis (about 600 mL at 150 mg/L). Ti Jacques and Sonson’s particle contents were consistent with their puree AIR yields, Galion’s was consistent with the hypothesis of material loss. The particle size distribution of the three accessions was also different (Figure 4). Ti Jacques and Sonson had opposite profiles while Galion had an intermediate distribution (Figure 4a). Medium-sized particles (70 µm < approximated diameter < 250 µm) were prevalent in Ti Jacques puree, representing about 50% of the total particle population, while small particles (<70 µm) represented about 5% of the total population and coarse particles (>250 µm), 45%. Half of the total particle population of Ti Jacques had approximated diameters lower than 160 µm (Figure 4b). On the opposite, coarse particles were prevalent in Galion and Sonson puree (about 65% and 85% of the total particles, respectively). Sonson had the lowest particle content and 50% of these particles had approximated diameters higher than 900 µm (Figure 4b). These differences in particle size distributions between the three accessions were consistent with the differences in pulp cell sizes observed in histological analysis. Indeed, compared to the other accessions, Sonson mostly had bigger endocarp cells while Ti Jacques accession mostly had smaller endocarp cells. As the samples were all prepared in the same conditions, cell wall fragments from bigger cells were more likely to be larger than those from smaller cells, resulting in opposite particle size distribution profiles for Sonson and Ti Jacques. Galion’s intermediate particle number and particle size distribution were consistent with this correlation as its biggest endocarp cells were smaller than Sonson’s and it had fewer small cells than Ti Jacques.

Larger particles, identified as cell clusters, were reported to increase viscosity in plant cell suspensions at intermediate and concentrated domains of insoluble solid concentration compared to smaller particles (cell wall fragments and individual cells) [26]. Ti Jacques’ viscosity was the lowest (1445 ± 24 mPa·s), which complied with its lower content in coarse particles and its lower particles sizes. Galion had the highest viscosity (1806 ± 48 mPa·s, *p* < 0.05), corresponding to its high content in coarse particles and diversity of population sizes, even though polydispersity was reported with little impact on overall rheology compared with particle shape [26]. Despite its higher content in coarse particles, Sonson’s viscosity (1491 ± 64 mPa·s) was not significantly different from Ti Jacques’. This may be related to Sonson’s significantly lower particle content resulting in less hindrance toward the flow as well as a difference in serum composition. Serum phase was reported with little impact on concentrated suspensions’ rheology, but a lubricating effect was observed thanks to its composition, particularly to pectin [26]. This lubricating effect facilitates flowing and may explain Sonson’s lower viscosity. It was noted that the higher the WSF pectin Mw (Table 3), the higher the puree viscosity, as previously reported for apple pectin, in relation to a higher number of side chains in the higher-Mw sample [20].

Comparable viscosities were obtained on mamey puree from a previous harvest [9]. The average viscosity values obtained were high compared to other fruit purees, such as guava [17]. This suggests that mamey processing yields may be lower than with these fruits, particularly for processes including sieving. Indeed, sieving has been reported as the critical step in puree preparation, giving the lowest yield, with crushing in second place [9]. The yield in puree preparation could be increased by improving the crushing method, for instance, by crushing under vacuum, which would have a positive impact on sieving. Flash vacuum expansion could efficiently break pulp tissues and improve yields [27].

## 3. Materials and Methods

### 3.1. Chemicals and Reagents

Acetone (HPLC grade) and ethanol (96%) were from Sigma-Aldrich (Lyon, France). Other chemicals such as acids, basis and reaction buffers were of analytical grade.

HPLC-grade ultrapure water was generated in house with a Millipore (Molsheim, France) water purification system.

### 3.2. Standards

Standards used for quantitative analysis: methanol (≥99.9%, Carlo Erba, Val-de-Reuil, France) and acetic acid (≥99.8%, Sigma-Aldrich), inositol (99.5%, Sigma-Aldrich), D-(+)-galacturonic acid (>97.0%, Fluka BioChemika, Buchs, Switzerland).

Enzymes used for immunolabeling: galactanase (E-EGALN, lot 101001b), endo-arabinanase (E-EARAB, lot 111201c) and endoglucanase (E-CELTR, lot 110901a) (Megazyme). Pectin lyase was purified from the Peclyve commercial mixture [28].

### 3.3. Plant Material

The fruits of three mamey (*Mammea americana* L., Callophylaceae) accessions, Galion, Sonson and Ti Jacques, were harvested ripe according to usual practice, that is, by gentle wrist rotation, to detach the fruit from the trees and by picking up fallen fruits within 24 h. Twelve ripe fruits per accession were harvested from the FREDON (Fédération régionale de défense contre les organismes nuisibles) experimental orchard in Rivière Lézarde (IGN coordinates: 14.662359, −60.997607, Saint-Joseph, Martinique).

### 3.4. Preparation of Puree and Grated Endocarp

Ten out of the twelve fruits harvested per accession were peeled and sliced manually with a knife. The epicarp (skin), the mesocarp (white layer between the skin and the flesh) and the seeds were discarded. The flesh was crushed in puree in a Moulinex Masterchef 5000 mixer (Groupe SEB, Saint-Lô, France). The puree obtained was manually sieved on a 2 mm inox sieve and stored at −80 °C until analysis. Half of the two remaining fruits of each accession was peeled and grated on a domestic food processor for the preparation of alcohol-insoluble residue (see Section 3.5). Finally, slices were cut off the rest of each fruit for immunochemistry study (see Section 3.7).

### 3.5. Preparation of Alcohol-Insoluble Residue (AIR)

The AIR was extracted from puree and grated endocarp as previously described [29]. A total of 150 g of puree or 250 g of grated endocarp were boiled at 95 °C for 20 min in an appropriate volume of 96% ethanol to obtain a final ethanol concentration of 70%. The supernatant was eliminated by filtration, and successive washings with 70% ethanol were applied to the residue until no remaining carbohydrate was detected in the supernatant using a phenol test [30]. The residue was rinsed with 96% ethanol, then rinsed with acetone and the AIR was dried overnight in an oven at 40 °C. AIR yields were calculated using the starting weights of the puree and grated endocarp samples.

### 3.6. Cell Wall Immunohistochemistry

#### 3.6.1. Sample Preparation

**Figure 5:**
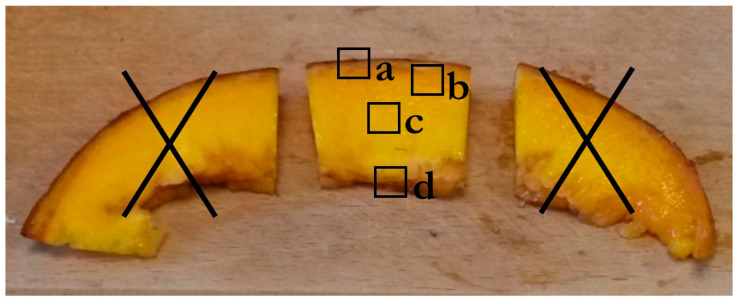
Central part of the slice with the areas used for microscopic study. Four areas delimited on the sample: a (epicarp/mesocarp); b (mesocarp/endocarp); c (endocarp); d (endocarp/internal epidermis).

The central part of the fruit slices was used for microscopy. Four areas were delimited on the sample: a (epicarp/mesocarp); b (mesocarp/endocarp); c (endocarp); and d (endocarp/internal edge) (Figure 5). Semi-thin sections were fixed in a mixture of 3% paraformaldehyde and 0.5% glutaraldehyde in phosphate buffer at pH 7.4 as previously described in [29]. They were embedded in LRW resin, and polymerization was realized for 2 days at 55 °C. Semi-thin sections (1 µm) were cut using an ultramicrotome (UC7, Leica Microsystems, Wetzlar, Germany) equipped with a diamond knife.

#### 3.6.2. Coloration for Observation under Macroscope

Semi-thin sections were bathed for 5 min in a 1% toluidine blue in 2% Na_2_CO_3_ solution, rinsed with demineralized water to stain the cell walls, then observed under white light. Other semi-thin sections restricted to the endocarp (c area, Figure 5) were stained for 2 to 3 min in 0.01% calcofluor in demineralized water to target β-linked cell wall polysaccharides. After briefly rinsing with demineralized water, the sections were observed at emission wavelength higher than 425 nm after excitation at 340–380 nm. Autofluorescence was checked under UV light and at the different wavelengths used for colorations.

Two sections by accession were observed, one from each fruit. The four areas of the sections were observed under a AZ100 NIKON microscope (Konan, Japan).

#### 3.6.3. Immunolabeling on Endocarps

Several primary antibodies and the carbohydrate binding module CBM3a were used to assess the spatial distribution of cell wall polysaccharides (Table 4).

Each labeling was performed on two semi-thin sections of the endocarp (c area, Figure 5) of each accession as previously described [29]. CBM3a and the primary antibody solutions were appropriately diluted (Table 4) in PBS+BSA 1% + 0.05% Tween20, supplemented by Tris/Ca/Na (20 mM) buffer for 2F4 antibody. Secondary antibody (GARAT Alexa 546 nm) was prepared in the same solution as the primary antibody. The observation was performed under a DMRB LEICA fluorescence microscope equipped with a DMRD camera system.

### 3.7. Cell Wall Chemical Composition

#### 3.7.1. Sample Preparation

Two grams of AIR from puree or grated endocarp were ultra-crushed in liquid nitrogen in a Spex 6700 cryogenic grinder (Spex. Industries, Metuchen, NJ, USA) and dried. A total of 500 mg of the ultra-crushed AIR powder was washed four times with 20 mL distilled water. No carbohydrate was detected by phenol-sulfuric acid test [30] in the last supernatant. The pooled supernatants were lyophilized then weighed and constituted the water-soluble fraction (WSF). The insoluble residues were lyophilized and weighed. They were referred to as water- and alcohol-insoluble residues (WAIRs). Prior to quantitative analysis AIR, WSF and WAIR were dried in a vacuum oven for 2 h at 40 °C.

#### 3.7.2. Quantitative Analysis of Sugars

Uronic acid (UA) content was determined by the colorimetric metahydroxydiphenyl method [32] performed using a Skalar auto-analyzer in H_2_SO_4_-tetraborate. The UA content was determined on the supernatants of hydrolyzed AIR and WAIR samples (H_2_SO_4_ 4 N, 100 °C, 2 h), after pre-hydrolysis (72% H_2_SO_4_, 25 °C, 30 min). The supernatants were recovered after centrifugation of the hydrolysis mixture (30,000× *g*, 20 °C, 10 min). The UA content was determined on WSF after de-esterification in 0.1 N NaOH for 30 min, and addition of 0.1 N HCl and water to obtain the dilution necessary for analysis. The calibration series was performed with galacturonic acid.

The neutral sugar (NS) compositions were determined by gas chromatography of alditol acetates [33] on the supernatants of AIR and WAIR with and without pre-hydrolysis, and of the hydrolyzed WSF without pre-hydrolysis. Analysis was performed on a Perkin Elmer Clarus^®^ 580 equipment (Perkinelmer Scientific, Villepinte, France). Inositol (5 g/L) was used as internal standard and the external standard solution was a mixture of 1 g/L rhamnose, fucose, arabinose, xylose, mannose, galactose, glucose and inositol.

Each sample was analyzed in duplicate and the results were expressed as percentage of dry weight of AIR, WSF or WAIR.

#### 3.7.3. Quantitative Analysis of Methanol and Acetic Acid

The methanol and acetic acid contents were determined by reverse-phase (RP)-HPLC analysis [34] on WSF samples, WAIR samples and ultra-crushed AIR powder. A mixture of methanol and acetic acid was prepared extemporaneously to be used as standard. Each sample was analyzed in duplicate and the results were expressed as percentage of dry weight of AIR, WAIR or WSF.

#### 3.7.4. Molecular Weight of Polysaccharides

Three milligrams of lyophilized WSF was suspended in 1 mL of water and added to 5 µL of LiOH 0.1 M. The mixture was filtered using a Whatman 0.45 µm PVDF filter (Grosseron, Coueron, France) and submitted to HPSEC (high-pressure size exclusion chromatography) analysis with refractive index (Viscotek VE 3580 RI detector, Malvern Instruments, Malvern, UK), multi-angle laser light scattering (low angle 7° and right angle) and viscosimetric detections (both via Viscotek 270 dual detector, Malvern Instruments). The samples were injected through a Shodex OHpak 805KB column equipped with an OHPak SB-G guard column (Thermo Fisher Scientific, Illirch, France). Pullulans were used as standards for system calibration. Molecular weight was obtained using the Omnisec 4.7 software (Malvern Instruments).

### 3.8. Rheological and Histological Characterization of Purees

Purees were analyzed after thawing at 4 °C. The viscosity of the fruit purees was determined using a viscometer at 20 °C (VT 500 HAAKE–SVDIN measuring system, Thermo Fisher Scientific, Illkirch, France).

The particles constituting the purees were analyzed using a QICPIC particle analyzer (SYMPATEC, Clausthal-Zellerfeld, Germany) equipped with a M9 optical module. The puree (150 mg) was suspended in 25 mL of milliQ water, sonicated with a probe (Bioblock Scientific, Illkirch, France) (2 min, pulse 2 s, 7 W), and prepared in a final volume of 1 L of milliQ water. The samples were analyzed for 300 s at room temperature under identical flow rate and sufficient magnetic stirring to avoid sedimentation. Under these conditions, the solutions were considered homogeneous and the same volume of each sample was analyzed. Image analysis with PAQXOS software (SYMPATEC, https://www.sympatec.com/en/particle-measurement/application-software/paqxos/, accessed on 18 February 2024) allowed for measuring the particles’ equivalent-projected circle (EQPC) diameter (hereafter named approximated diameter).

### 3.9. Statistical Analysis

The rheological and histological characterization assays were performed in triplicate for each accession. Results were expressed as means values ± standard deviation (*n* = 3). The data were analyzed for significant differences between accessions (*p*-value < 0.05) by means of a Kruskal–Wallis test on Excel^®^ (version 2301).

## 4. Conclusions

This first insight into the cell walls of pulp from three mamey accessions selected in Martinique shows how their composition is impacted by processing, and how their content relates to the rheological behaviors of the purees. Pectins are the main components of the mamey cell wall and vary in size, composition and degree of esterification depending on the accessions, where the insoluble fraction is always more important than the soluble fraction. Pectin predominantly consisted of highly methyl-esterified homogalacturonans with some acetyl esters. It embedded cellulose in a matrix comprising a low amount of xyloglucan as the main hemicellulose. Processing mamey pulp into puree mainly reduced highly methylated homogalacturonan contents and resulted in three purees with significantly different viscosities. These differences were consistent with particle number and size distribution and with water-soluble pectin properties peculiar to each accession. This study reports valuable knowledge to guide future mamey processing strategies.

## Figures and Tables

**Table 1:** Yields and chemical composition of AIR from fresh mamey pulp and mamey puree.

Sample	Accession	Yield ^a^	Proteins ^b^	Ashes ^b^	UA ^b^	Methanol (DM) ^b^	Acetic Acid ^b^	Cel Glc ^c^	Non-Cel Glc ^d^	Ara ^e^	Gal ^e^	Xyl ^e^	Man ^e^	Rha ^e^	Fuc ^e^
Fresh pulp	Galion	3.4 ± 0.1	11.2 ± 0.3	1.1 ± 0.1	30.8 ± 0.1	4.0 ± 0.2 (73.1 ± 2.8)	2.7 ± 0.1	16.3 ± 0.6	0.7 ± 0.1	8.8 ± 0.3	6.1 ± 0.5	2.9 ± 0.1	1.4 ± 0.0	1.1 ± 0.1	0.4 ± 0.0
	Ti Jacques	3.5 ± 0.0	7.8 ± 0.3	1.0 ± 0.1	28.8 ± 0.1	3.7 ± 0.1 (72.5 ± 1.2)	2.9 ± 0.0	15.1 ± 0.8	0.6 ± 0.0	9.1 ± 0.0	6.3 ± 0.2	3.0 ± 0.3	1.3 ± 0.1	1.0 ± 0.1	0.4 ± 0.0
	Sonson	3.0 ± 0.4	8.4 ± 0.3	1.2 ± 0.1	33.6 ± 0.9	4.6 ± 0.0 (76.7 ± 0.2)	2.7 ± 0.1	14.8 ± 0.1	0.9 ± 0.1	8.2 ± 0.8	4.4 ± 0.8	2.5 ± 0.1	1.7 ± 0.2	0.9 ± 0.0	0.4 ± 0.0
Puree	Galion	2.3	12.3 ± 0.6	1.9 ± 0.0	29.0 ± 0.0	3.5 ± 0.0 (67.4 ± 0.1)	2.6 ± 0.0	17.6	0.8 ± 0.1	9.1 ± 0.0	6.3 ± 0.0	2.8 ± 0.0	1.5 ± 0.0	1.2 ± 0.0	0.4 ± 0.0
	Ti Jacques	3.8	10.7 ± 1.7	1.3 ± 0.1	26.0 ± 0.1	3.1 ± 0.1 (66.2 ± 1.0)	2.8 ± 0.1	18.6	0.8 ± 0.1	9.0 ± 0.1	6.8 ± 0.0	3.3 ± 0.0	1.6 ± 0.0	1.3 ± 0.1	0.4 ± 0.0
	Sonson	3.1	10.8 ± 0.5	1.1 ± 0.0	32.7 ± 0.3	4.2 ± 0.1 (72.1 ± 1.6)	2.5 ± 0.1	19.3	0.8 ± 0.1	8.6 ± 0.1	6.1 ± 0.2	2.6 ± 0.1	1.8 ± 0.0	1.2 ± 0.1	0.5 ± 0.0

^a^ AIR yield is expressed as g/100 g of fresh pulp or puree. ^b^ Expressed as g/100 g of dry weight. DM: degree of methyl-esterification expressed as moles of methanol per 100 moles of UAs (uronic acids). ^c^ Cel Glc: cellulosic glucose, expressed as g/100 g of dry weight, deduced from the difference between glucose content measured with and without acid pre-hydrolysis. ^d^ Non-Cel Glc: non-cellulosic glucose, expressed as g/100 g of dry weight. ^e^ Ara: arabinose; Gal: galactose; Xyl: xylose; Man: mannose; Rha: rhamnose; Fuc: fucose; maximum value obtained from analysis with or without acid pre-hydrolysis, expressed as g/100 g of dry weight.

**Table 2:** Yields and chemical composition of WSF and WAIR from fresh mamey pulp and mamey puree.

Accession	Sample	Yield ^a^	UA ^b^	Methanol (DM) ^b^	Acetic Acid ^b^	Cel Glc ^c^	Non-Cel Glc ^d^	Ara ^e^	Gal ^e^	Xyl ^e^	Man ^e^	Rha ^e^	Fuc ^e^	UA/Rha
**WSF**														
**Galion**	Fresh pulp	20.7 ± 0.4	73.4 ± 2.5	9.2 ± 0.8 (68.4 ± 3.4)	2.2 ± 0.2	nd	0.9 ± 0.1	6.3 ± 0.4	3.4 ± 0.3	1.8 ± 0.0	<dL	1.5 ± 0.1	0.2 ± 0.0	41.2 ± 0.2
	Puree	19.0	70.9 ± 1.7	9.1 ± 0.2 (70.3 ± 0.0)	2.4 ± 0.0	nd	0.8 ± 0.0	5.7 ± 0.1	3.1 ± 0.0	1.6 ± 0.0	<dL	1.4 ± 0.0	0.2 ± 0.0	42.0
**Ti Jacques**	Fresh pulp	17.4 ± 0.7	81.6 ± 3.6	9.7 ± 0.2 (65.7 ± 4.2)	1.9 ± 0.1	nd	0.8 ± 0.1	6.0 ± 0.0	3.0 ± 0.1	1.3 ± 0.1	<dL	1.2 ± 0.1	0.2 ± 0.0	56.9 ± 1.5
	Puree	11.8	70.5 ± 1.2	8.9 ± 0.3 (69.7 ± 2.3)	1.9 ± 0.0	nd	0.9 ± 0.0	6.3 ± 0.5	3.1 ± 0.2	1.5 ± 0.1	0.3 ± 0.0	1.5 ± 0.0	0.2 ± 0.0	38.5
**Sonson**	Fresh pulp	22.7 ± 0.4	73.7 ± 2.3	9.1 ± 0.5 (67.5 ± 1.6)	1.6 ± 0.1	nd	0.9 ± 0.0	5.4 ± 1.8	2.2 ± 0.3	0.8 ± 0.2	<dL	1.2 ± 0.4	0.2 ± 0.1	56.3 ± 16.0
	Puree	19.0	66.4 ± 0.3	9.6 ± 0.1 (79.2 ± 0.7)	1.9 ± 0.0	nd	1.0 ± 0.1	5.7 ± 0.2	3.0 ± 0.1	0.9 ± 0.1	0.3 ± 0.0	1.2 ± 0.0	0.2 ± 0.0	47.0
**WAIR**														
**Galion**	Fresh pulp	75.2 ± 0.4	24.5 ± 0.6	2.6 ± 0.0 (58.5 ± 1.2)	2.5 ± 0.0	35.8 ± 1.0	1.0 ± 0.2	11.3 ± 0.7	8.4 ± 1.1	4.6 ± 0.0	2.0 ± 0.1	1.8 ± 0.1	0.7 ± 0.0	11.0 ± 0.8
	Puree	77.8	23.7 ± 0.4	2.4 ± 0.0 (56.7 ± 1.0)	2.5 ± 0.1	34.8	0.9 ± 0.0	11.0 ± 0.0	8.5 ± 0.0	4.2 ± 0.0	2.0 ± 0.0	1.8 ± 0.1	0.7 ± 0.0	11.1
**Ti Jacques**	Fresh pulp	79.5 ± 0.7	23.4 ± 1.2	2.7 ± 0.2 (63.8 ± 0.3)	2.9 ± 0.1	38.7 ± 0.7	0.9 ± 0.0	12.3 ± 0.2	9.5 ± 0.5	5.3 ± 0.3	2.0 ± 0.0	1.9 ± 0.1	0.7 ± 0.0	10.3 ± 1.0
	Puree	76.8	18.8 ± 0.0	1.9 ± 0.0 (54.8 ± 1.1)	2.7 ± 0.1	33.1	1.0 ± 0.0	11.2 ± 0.3	9.2 ± 0.5	4.7 ± 0.3	2.1 ± 0.2	1.7 ± 0.3	0.6 ± 0.1	9.2
**Sonson**	Fresh pulp	73.6 ± 0.7	24.6 ± 0.4	2.8 ± 0.1 (62.9 ± 1.3)	2.6 ± 0.0	40.2 ± 0.0	1.3 ± 0.0	11.7 ± 0.7	7.0 ± 1.5	4.8 ± 0.0	3.0 ± 0.3	1.6 ± 0.1	0.8 ± 0.0	13.2 ± 1.0
	Puree	75.8	26.0 ± 0.2	3.0 ± 0.1 (62.8 ± 1.6)	2.6 ± 0.1	40.9	0.9 ± 0.1	11.2 ± 0.1	8.5 ± 0.1	4.4 ± 0.0	3.7 ± 1.4	1.8 ± 0.0	0.8 ± 0.0	12.1

^a^ Yield is expressed as g/100 g of AIR. ^b^ Expressed as g/100 g of dry weight. DM: degree of methyl-esterification expressed as moles of methanol per 100 moles of UAs. ^c^ Cel Glc: cellulosic glucose, expressed as g/100 g of dry weight, deduced from the difference between glucose content measured with and without acid pre-hydrolysis. ^d^ Non-Cel Glc: non-cellulosic glucose, expressed as g/100 g of dry weight. ^e^ Ara: arabinose; Gal: galactose; Xyl: xylose; Man: mannose; Rha: rhamnose; Fuc: fucose; maximum value obtained from analysis with or without acid pre-hydrolysis, expressed as g/100 g of dry weight.

**Table 3:** WSF molecular weight, viscosity and side chain composition ratio.

Sample	Accession	Mw ^a^	IV ^b^	I ^c^	(Ara + Gal)/Rha ^d^
Fresh pulp	Galion	350	8.5	2.3	6.7
	Ti Jacques	350	11.0	2.1	7.8
	Sonson	334	10.3	1.8	7.1
Puree	Galion	392	9.0	2.3	6.5
	Ti Jacques	295	8.1	2.3	6.4
	Sonson	355	10.3	1.9	7.6

^a^ Weight-average molecular weight (Mw) expressed as kDa. ^b^ Intrinsic viscosity (IV) expressed as dL/g; ^c^ I: polydispersity index (=Mw/Mn with Mn: number-average molecular weight); ^d^ (Ara + Gal)/Rha: (Arabinose + galactose)/rhamnose ratio.

**Table 4:** Labeling conditions: sample preparation and probes used [31].

Probes	Suppliers	Origin	Targets	Dilution	Sample Pretreatments ^a^
**INRA-RU1**	INRAE Nantes [31]	Mouse	Rhamnogalacturonan I	1/3	Alkali: incubation in 50 nM NaOH for 20 min at 4 °C
**LM5**	PlantProbes	Rat	(1–4)-β-D-galactan	1/10	Alkali + Enzymatic: incubation overnight at 38 °C Mixture of galactanase (50 U/mL) and endo-arabinanase (50 U/mL)
**LM6**			Linear (1–5)-α-L-arabinan	1/10	
**LM19**			Weakly methyl-esterified homogalacturonan	1/10	Enzymatic: - Pectin lyase: incubation overnight at 40 °C
**LM20**			Strongly methyl-esterified homogalacturonan	1/10	
**2F4**			Weakly methyl-esterified homogalacturonan chelated by Ca^2+^	1/5	None
**LM15**			Xyloglucan	1/5	Enzymatic: Pectin lyase: incubation overnight at 40 °C Mixture of pectin lyase and endoglucanase (50 U/mL): incubation overnight at 38 °C
**LM21**			Mannan	1/5	
**CBM3a**		*Clostridium thermocellum*	Crystalline cellulose	1/975	

^a^ After each pretreatment, semi-thin sections were intensively rinsed with demineralized water.

## Data Availability

Data are contained within the article.
